# Wild Type and Mutant 2009 Pandemic Influenza A (H1N1) Viruses Cause More Severe Disease and Higher Mortality in Pregnant BALB/c Mice

**DOI:** 10.1371/journal.pone.0013757

**Published:** 2010-10-29

**Authors:** Kwok-Hung Chan, Anna J. X. Zhang, Kelvin K. W. To, Chris C. S. Chan, Vincent K. M. Poon, Kunyuan Guo, Fai Ng, Qi-Wei Zhang, Virtual H. C. Leung, Annie N. Y. Cheung, Candy C. Y. Lau, Patrick C. Y. Woo, Herman Tse, Wailan Wu, Honglin Chen, Bo-Jian Zheng, Kwok-Yung Yuen

**Affiliations:** 1 Department of Microbiology, University of Hong Kong, Hong Kong Special Administrative Region, China; 2 Research Centre of Infection and Immunology, University of Hong Kong, Hong Kong Special Administrative Region, China; 3 State Key Laboratory for Emerging Infectious Diseases, University of Hong Kong, Hong Kong Special Administrative Region, China; 4 Department of Pathology, University of Hong Kong, Hong Kong Special Administrative Region, China; La Jolla Institute of Allergy and Immunology, United States of America

## Abstract

**Background:**

Pregnant women infected by the pandemic influenza A (H1N1) 2009 virus had more severe disease and higher mortality but its pathogenesis is still unclear.

**Principal Findings:**

We showed that higher mortality, more severe pneumonitis, higher pulmonary viral load, lower peripheral blood T lymphocytes and antibody responses, higher levels of proinflammatory cytokines and chemokines, and worse fetal development occurred in pregnant mice than non-pregnant controls infected by either wild type (clinical isolate) or mouse-adapted mutant virus with D222G substitution in hemagglutinin. These disease-associated changes and the lower respiratory tract involvement were worse in pregnant mice challenged by mutant virus. Though human placental origin JEG-3 cell line could be infected and proinflammatory cytokines or chemokines were elevated in amniotic fluid of some mice, no placental or fetal involvement by virus were detected by culture, real-time reverse transcription polymerase chain reaction or histopathological changes. Dual immunofluorescent staining of viral nucleoprotein and type II alveolar cell marker SP-C protein suggested that the majority of infected alveolar epithelial cells were type II pneumocytes.

**Conclusion:**

The adverse effect of this pandemic virus on maternal and fetal outcome is largely related to the severe pulmonary disease and the indirect effect of inflammatory cytokine spillover into the systemic circulation.

## Introduction

The pandemic influenza A(H1N1) 2009 virus caused similar spectrum of illness as seasonal influenza virus except for more severe diseases in younger adults with little cross-reactive neutralizing antibody [1], [2], [3], [4]. Besides extremes of age and underlying medical illness, obesity and pregnancy were also significant risk factors for severe infection by this pandemic virus [5],[6],[7],[8],[9]. Pregnancy is a well-known risk factor for severe disease in seasonal influenza [10]. Other viral infections, such as chickenpox, have been shown to be more likely to cause fatality in pregnant women than non-pregnant adults [11]. Despite many reports on severe influenza in pregnancy, few immunological and histopathological studies on its pathogenesis were performed. Moreover reports on the virulence of this pandemic virus in mice model were contradictory [2],[12]. Recently we and others showed that the substitution of glutamate by glycine at position 222 of the viral hemagglutinin (D222G by H1 numbering or D225G by H3 numbering) was found to be significantly more frequent in patients with severe pandemic influenza H1N1 [13],[14],[15],[16],[17],[18]. This mutant virus often existed as quasispecies, and had increased predilection for the lower respiratory tract [13]. Furthermore the mutant virus was more virulent than the parental clinical isolate in BALB/c mice [19]. To understand the pathogenesis of this novel virus in pregnancy, we analyze the chemokine and cytokine profiles, viral load and histopathological changes in placental cell line and BALB/c mice infected by the wild type pandemic influenza A(H1N1) 2009 virus, which is a clinical isolate from a patient with mild disease, and a D222G mutant, which can cause severe clinical outcome.

## Materials and Methods

### Virus strains

The human clinical isolate influenza virus H1N1, HK/415742/09 (wild type) and mouse-adapted strain HK/415742/09-Mut, which has a single D222G substitution in hemagglutinin (HA) gene, were purified by plaque forming assay in Madin-Darby canine kidney (MDCK) cells. The purified viruses were grown in embryonated eggs for animal experiments and MDCK cells for cell culture experiments as reported previously [19]. After titer determination, the cultured viruses were aliquoted and kept at −80°C till use.

### Virus-turkey erythrocyte binding assay

Virus-turkey erythrocyte binding assay was performed as previously described with minor modifications [20]. Briefly, turkey erythrocytes were treated with different concentrations of *Vibrio cholerae* neuraminidase (Sigma, St. Louis, USA) for 60 min at 37°C to remove the 2,3-linked sialic acid. The erythrocytes were washed twice with phosphate buffered saline (PBS) and then diluted to 2% (v/v) erythrocytes solutions with PBS. The 2% erythrocyte solution (25 µl) was mixed with 8 HA unit of influenza viruses (100 µl) and incubated at room temperature for 60 min. Hemagglutination was measured and data expressed as the maximal concentration of neuraminidase that allowed for full hemagglutination.

### Viral infection in human placental cell line

The wild type and mutant viruses were inoculated into human choriocarcinoma cell line JEG-3 (HTB-36, ATCC, Rockville, MD) at 1 multiplicity of infection (MOI) per cell and incubated at 37°C. Supernatants were collected at 6, 24, 72 and 120 hours post-infection for the determination of viral loads by real time reverse-transcription polymerase chain reaction (RT-PCR) and antigen expression by immunofluorescence method as reported previously [21], while infected cells were harvested at 1, 3 and 6 hours post-infection for the detection of proinflammatory cytokines and chemokines, by real time RT-PCR in triplicate for each sample as reported previously [22].

### Viral infection in pregnant and non-pregnant mice

Pregnant (11-weeks old) and age-matched non-pregnant female BALB/c mice were kept in biosafety level-3 housing and given access to standard pellet feed and water ad libitum. All experimental protocols followed the standard operating procedures of the approved biosafety level-3 animal facilities and were approved by the Animal Ethics Committee (committee on the use of live animals in teaching & research CULATR No. 1929_09) [23]. Pregnant mice at the gestational ages of 12–14 days and non-pregnant mice were intranasally inoculated with 2×10^6^ plaque forming units (PFU) of wild type virus and 150 PFU of mutant virus (Table 1), respectively, after anesthesia with ketamine (100 mg/kg) and xylazine (10 mg/kg) by intraperitoneal injection. Signs of the disease and survival were observed daily till day 21 post-infection. Eight (4 per experiment done in duplicate) mice per group were sacrificed for collections of blood, lung, brain, kidney, liver, spleen, placenta, fetus and amniotic fluid specimens at day 3 and 6 post-infection, respectively. For the pregnant groups, four placentas and 4 fetuses were also collected from each pregnant mouse, of which half were frozen immediately and the other half were fixed in formalin for further study.

**Table 1:** Viral challenge and sample collections in pregnant and non-pregnant mice.

	Challenge by wild type virus			Challenge by mutant virus		
	Total numbers	Survival observation	Sampling numbers	Total numbers	Survival observation	Sampling numbers
Pregnant	24	8	16	25	9	16
Non-pregnant	25	9	16	25	9	16

Four groups of the mice (12-13 mice/group) were intranasally inoculated with wild type or mutant viruses. Four groups of mice were sampled at day 3 and 6 post-infection, respectively. The experiment was duplicated. This table showed the total numbers of mice in these two experiments.

### Viral titration and viral load detection

Titers of released virus in lung homogenates were determined by plaque assays, whereas viral RNA in lung tissues, placenta and foetus was quantified by real-time RT-PCR as reported previously [19],[19,23]. Plaque forming assays were not performed if real-time RT-PCR is not negative for viral RNA.

### Detections of cytokines and chemokines

Levels of pro-inflammatory cytokines and chemokines in lung homogenates, serum and amniotic fluid were determined by ELISA as described previously [19],[19,23]. The samples were also collected from uninfected pregnant and non-pregnant mice as negative controls.

### Histopathological examinations

Mouse lung, brain, kidney, liver, spleen, placenta and fetus were immediately fixed in 10% formalin after sampling and embedded in paraffin. Tissue sections of 5 µm were stained with hematoxylin and eosin (H&E) [23]. Expression of influenza A viral nucleoprotein (NP) in lung, placenta and fetal tissues was examined by immunohistochemical staining. The tissue sections were deparaffinized and rehydrated, followed by blocking with 1% bovine serum albumin in PBS to minimize non-specific staining. The sections were incubated with mouse anti-influenza nucleoprotein monoclonal antibody (HB65, ATCC) at 1∶5000 dilution overnight at 4°C. After washing for 3 times with PBS, the sections were incubated with a secondary antibody with biotin conjugated goat anti-mouse IgG (Calbiochem, Darmstadt, Germany) at 1∶2000 dilution for 30 minutes at room temperature. Streptavidin/peroxidase complex reagent (Vector Laboratories, Burlingame, CA, USA) was then added and incubated at room temperature for 30 min. Color development was performed with diaminobenzidine (DAB, Vector Laboratories, Burlingame, CA, USA) and the images were captured by Nikon 80*i* imaging system with Spot-advance computer software. To determine the type of virus infected cells in mouse lung alveoli, viral NP protein and type II alveolar cell marker SP-C protein were examined for their co-localization within the infected cells. Briefly, after blocking with 1% bovine serum albumin in PBS, the tissue sections were incubated with a mixture of mouse anti-influenza nucleoprotein monoclonal antibody and rabbit anti-mouse SP-C antibody (Santa Cruz Biotechnology, Santa Cruz, CA USA) at 1∶200 dilution. Fluorescence conjugated secondary antibodies, i.e. Texas Red conjugated donkey anti-rabbit IgG and FITC conjugated donkey anti-mouse IgG (JacksoniImmunoResearch, West Grove, PA, USA) were then added and incubated at room temperature for 30 min. The sections were mounted in vectashield mounting medium with DAPI and analyzed under Nikon 80*i* fluorescent microscope imaging system with Spot-advance computer software.

### Peripheral T lymphocyte count and antibody level

Peripheral T cells in heparinized blood samples collected on day 3 and day 6 post-infection from infected and non-infected mice were stained with fluorescein-labeled anti-mouse CD3 (Cy5), CD4 (FITC) and CD8a (PE) monoclonal antibodies (BioLegend, San Diego) and fixed with 4% *p*-formaldehyde overnight. The fixed blood cells were analyzed by flow cytometer (FACSCaliber, BD, USA) and the serum antibody level was detected as described previously [23].

### Statistical Analysis

Survival of mice was analyzed by the Kaplan-Meier method and Log-rank test using SPSS 17.0 for Windows (SPSS Inc., Chicago, IL), whereas viral load, cytokine and chemokine profiles were calculated by Student's t-test. Results were considered significant at *P*<0.05. Results of the virus-turkey erythrocyte binding assay was analyzed by one-way ANOVA using the mean square in the ANOVA to estimate of variability.

## Results

### Infection of placental cell line by both clinical isolate and mouse adapted mutant viruses

**Figure 1:**
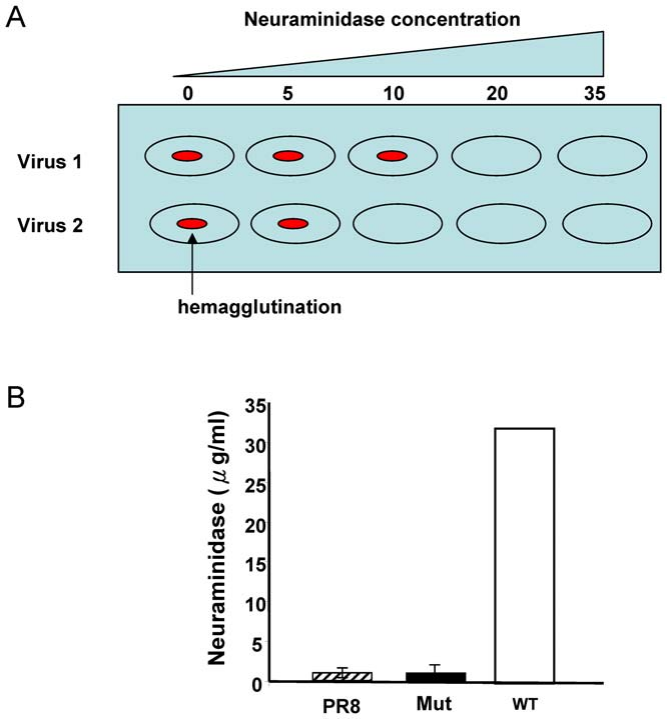
Virus-turkey erythrocyte receptor binding avidity assay. Influenza virus cellular receptor binding affinity was tested with turkey erythrocytes pre-treated with *Vibrio cholerae* neuraminidase which mainly removes 2, 3-linked sialic acids as described in the Materials and Methods. A) Illustration of assay for receptor binding avidity. B) Receptor binding avidity of mut and wt viruses. Data are representative of three independent experiments. Data was analyzed by one-way ANOVA using the mean square in the ANOVA to estimate of variability. P-value<0.001. PR8, A/Puerto Rico/8/34; Mut, mutant virus; WT, wild type virus.

Two isolates were used in this study. One isolate, HK/415742/09, was characterized from the first patient with pandemic influenza A(H1N1) 2009 virus infection in Hong Kong in May 2009 [24] and the other isolate was a mutant virus, HK/415742/09-Mut after mouse adaptation of the clinical isolate. Mouse adapted virus was found to have increased virulence in mice [19]. Genetic analysis of mutant virus found that change of virulence may be attributed to the D222G substitution in the HA gene. In a binding assay using turkey erythrocytes treated with neuraminidase which mainly removed 2,3-linked sialic acids, a 32-fold higher concentration of neuraminidase was necessary to inhibit the hemagglutination of turkey erythrocytes by wild type virus as compared to mutant virus (Fig. 1). The results suggested that the mutant virus preferably binds to α-2,3 linked sialic acid receptor, a receptor of influenza A virus which is more abundantly expressed in lower respiratory tract.

**Figure 2:**
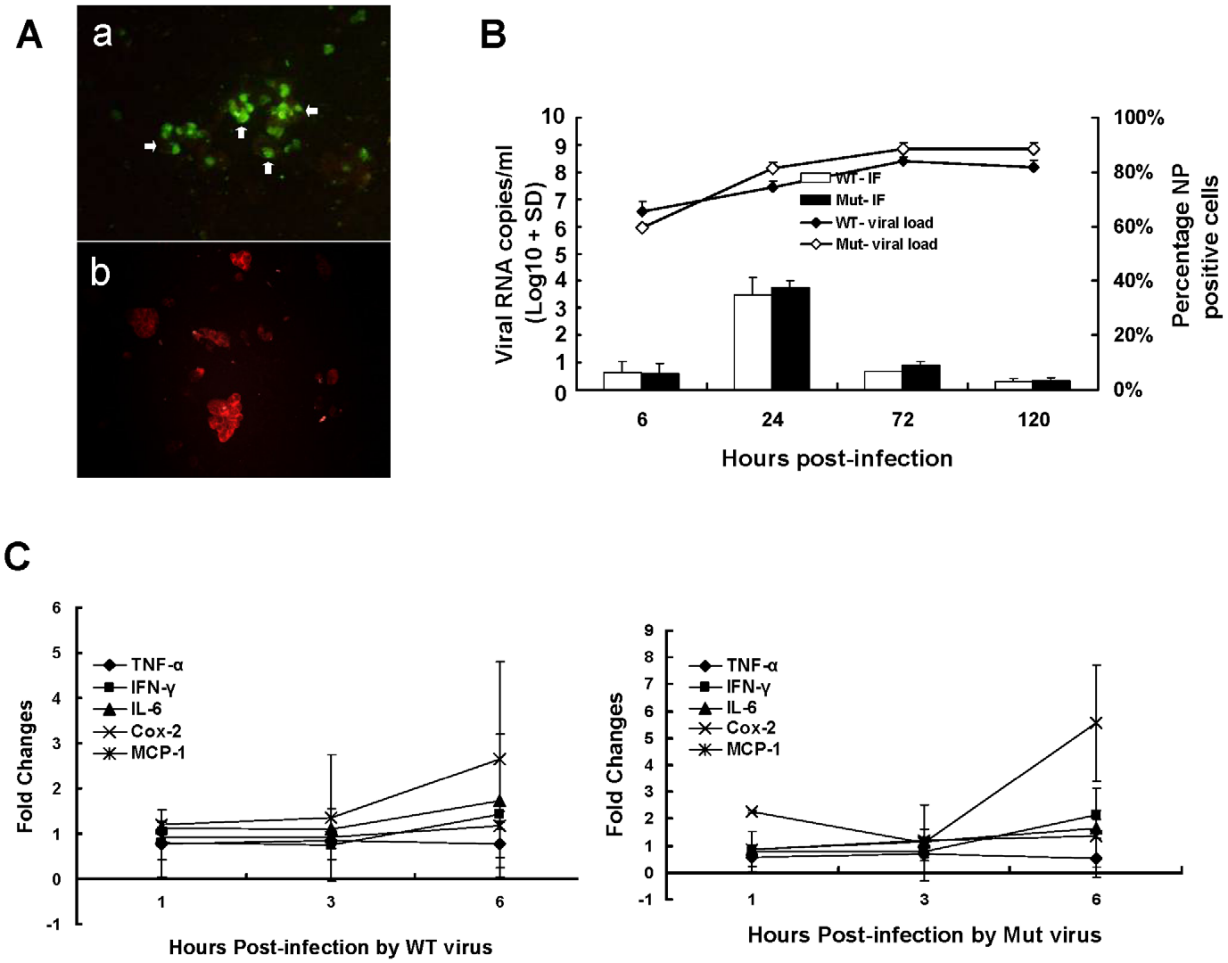
Viral infection in JEG-3 cells. A. Direct immunofluorescent staining of JEG-3 cell lines using anti-influenza nucleoprotein (NP) antibody showed viral NP expression in wild type virus-infected cells (a), but not in negative control of uninfected cells (b). B. Viral load (viral RNA copies/ml) determined by real-time RT-PCR and percentage of JEG-3 cells with immunofluorescent stained viral NP after infection by wild type (WT) or mutant (Mut) virus were shown. Error bar indicates ± standard deviation (SD) from triplicate experiments. C. Pro-inflammatory cytokine and chemokine expressions in JEG-3 cells infected with wild type or mutant virus determined by real-time RT-PCR at indicated time-points. Error bar indicates ± SD from 3 different experiments. TNF-α: tumor necrosis factor-α; IFN-γ: interferon-γ; IL-6: interleukin-6, Cox-2: cyclooxgenase 2; MCP-1: monocyte chemotactic protein-1.

A placental cell line, JEG-3 cells, derived from human choriocarcinoma, was used to examine infection and replication of the pandemic H1N1 virus. Our results showed that JEG-3 cells can be infected by both wild type and mutant viruses (Fig. 2A). Though no obvious cytopathic effects appeared after more than 5 days of cultures, viral load in culture supernatant increased about 2.5 logs for both wild type and mutant viruses after 24 hours of infection (Fig. 2B). Proinflammatory cytokine and chemokine gene expressions increased 0.5 to 6 folds from baseline levels at 6 hours post-infection (Fig. 2C). Cox 2 gene was highly induced by the mutant virus when compared with the wild type. The results suggest a potential of this virus in causing infection and inflammation of the placenta in human.

### Infected pregnant mice exhibited significantly higher mortality

**Figure 3:**
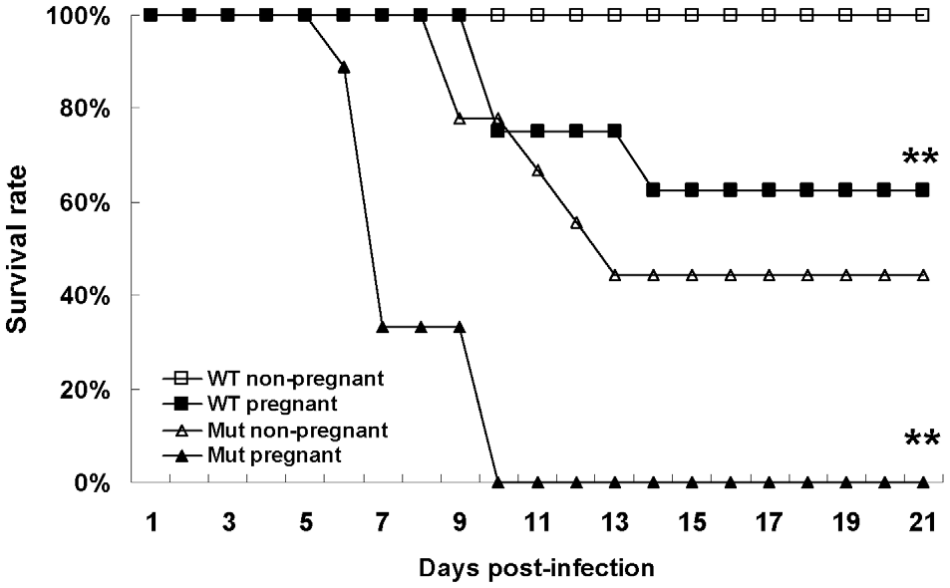
Survival rates of infected mice. Pregnant and non-pregnant BALB/c mice infected with wild type (WT) and mutant (Mut) viruses and their survivals were recorded in indicated days. ** indicates significant difference (*P*<0.001) between pregnant and non-pregnant mice.

Intranasal inoculation with 2×10^6^ PFU of wild type virus did not cause lethal infection in non-pregnant BALB/c mice (Fig. 3). Although infected mice showed ruffled fur and labored breathing for a few days, they later recovered and survived for 21 days. In contrast, when pregnant mice were challenged with the same dose of wild type virus, only 62.5% of infected pregnant mice survived, with a mean survival time of 17 days. In another challenge experiment, intranasal inoculation of 150 PFU of mutant virus caused 44.4% and 100% death in non-pregnant mice and pregnant mice, respectively. These results showed that either low pathogenic wild type virus or highly pathogenic mutant virus carrying a D222G substitution in HA [19] resulted in significantly higher mortality in pregnant mice than in non-pregnant mice (*P*<0.001).

### Infected pregnant mice showed higher levels of viral replication in the lungs

**Figure 4:**
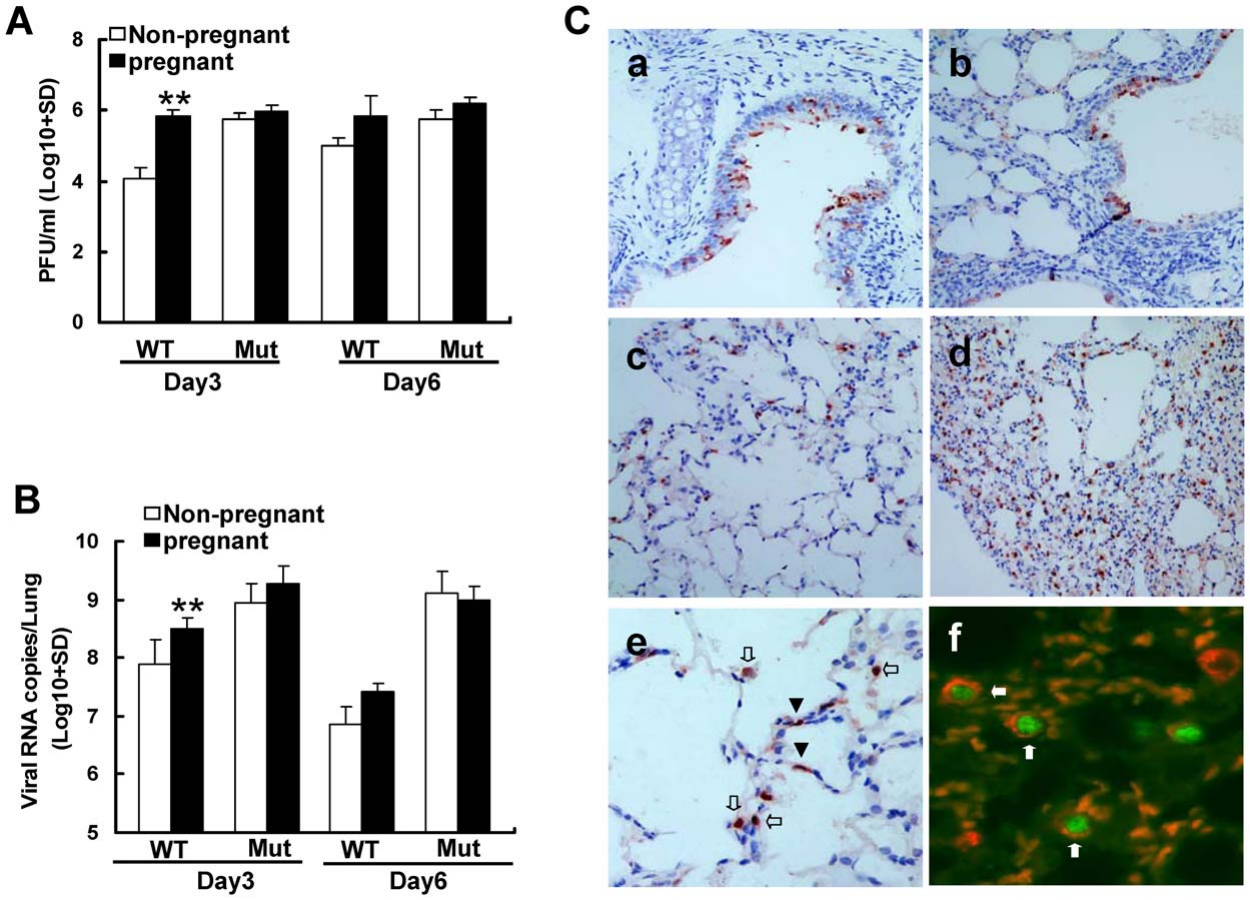
Viral infection and replication in lungs. A. Virus titers in lung homogenates collected from mice infected with wild type (WT) or mutant (Mut) virus on indicated days post-infection were measured by plaque forming assay. ** indicates significant differences (*P*<0.001) between pregnant and non-pregnant mice. B. Viral RNA copies in the same lung homogenates were also determined by real-time RT-PCR. * indicates significant differences (*P*<0.001) between pregnant and non-pregnant mice. C. Immunohistochemical staining for influenza viral nucleoprotein (NP) protein in lung sections of infected mice. Representative images of lung sections from wild type virus infected bronchial epithelium of non-pregnant (a) and pregnant (b) mice, and mutant virus infected alveoli of non-pregnant (c) and pregnant (d) mice. Original magnification: 100×. The NP positive cells were morphologically identified to be type I alveolar cells (few) indicated by arrow heads and type II alveolar cells (majority) indicated by arrows (e). Original magnification: 200×. Dual-labeling of mouse lung type II alveolar cells with anti-NP antibody conjugated with FITC and anti-SP-C antibody conjugated with Texas red further confirmed that the virus mainly infected type II alveolar cells (f). Arrows indicates NP and SP-C double positive alveolar cells. Original magnification: 400×.

Viral titers (Fig. 4A) and viral loads (Fig. 4B) in lung homogenates of mice infected by wild type were significantly higher in pregnant mice than in non-pregnant mice at day 3 post-infection (*P*<0.01). Furthermore, viral loads in lung tissues of both pregnant and non-pregnant mice infected with the mutant virus were also significantly higher than those infected by wild type virus (P<0.05, Fig. 4A & 4B) consistent with our previous report that D222G mutant is more likely to replicate in the lower respiratory tract tissues [19]. No evidence of viral infection was found in brain, liver, kidney, spleen, placenta and fetus by quantitative RT-PCR (data not shown).

Immunohistochemical staining of lung sections showed that viral NP was expressed mainly in bronchial and alveolar epithelial cells (Fig. 4C). The number of NP positive cells was much lower in non-pregnant mice infected with both wild type (Fig. 4Ca) and mutant (Fig. 4Cc) than in infected pregnant mice (Fig. 4Cb & Cd). NP positive cells were more abundant in lungs from mice infected by mutant virus (Fig. 4Cc & Cd) than that of mice infected by wild type virus (Fig. 4Ca & Cb). These findings are consistent with the histological severity of pneumonitis in infected mice. Furthermore, in mice infected with wild type virus, NP positive cells were mainly distributed in bronchial epithelial cells and just a few sporadic alveolar cells (Fig. 4Ca & Cb). While with mutant virus infection, abundant NP positive alveolar cells were found in addition to the positive bronchial epithelial cells (Fig. 4Cc & Cd). Morphologically, most NP positive alveolar cells were type II pneumocytes with occasional type I pneumocytes (Fig. 4Ce). Dual immunofluorescent staining of viral NP and type II pneumocyte marker SP-C protein indeed confirmed that most of the NP positive pneumocytes were type II cells (Fig. 4Cf).

### Infected pregnant mice exhibited more severe pneumonia

**Figure 5:**
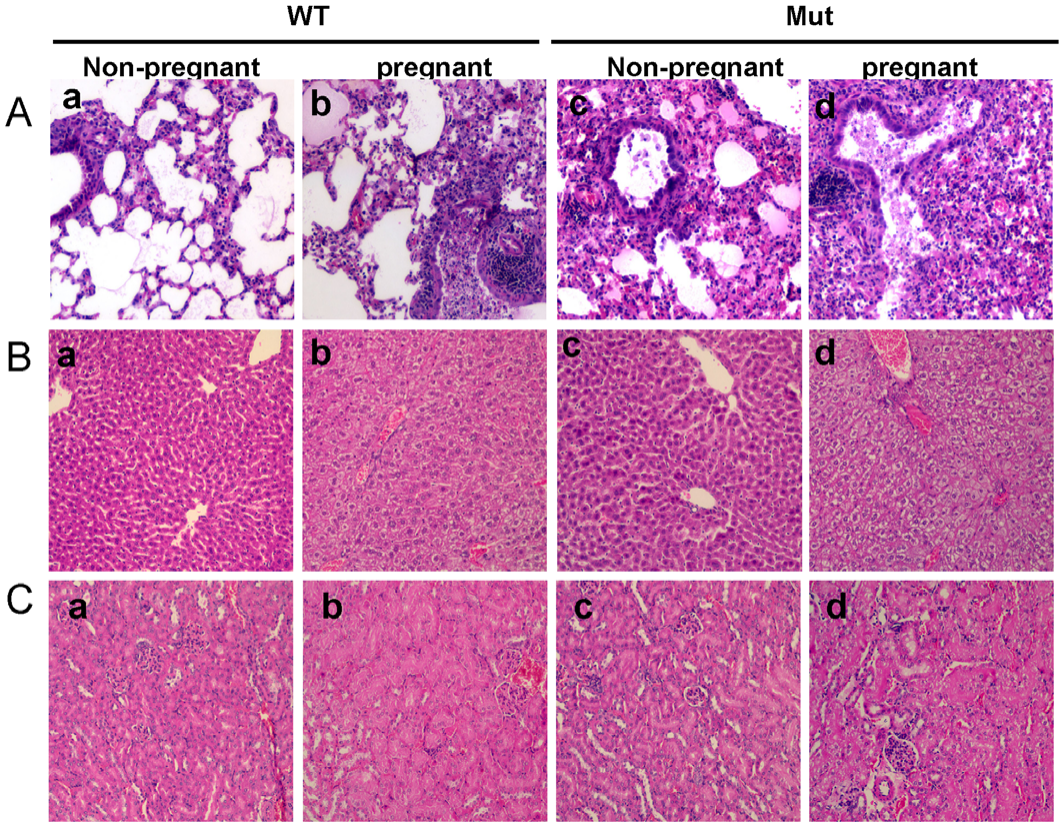
Histopathological changes in lungs, livers and kidneys of infected mice. Representative H&E stained sections showing changes in lung, liver and kidney tissues of mice infected with wild type (a & b) or mutant virus (c & d). A. Various degrees of interstitial bronchitis, epithelial necrosis, alveolitis and alveolar edema were found in lung tissues. B. Various degrees of degeneration were shown in liver cells. C. Degeneration of renal tubular epithelial cells was found in kidney tissues. Original magnification: 200×.

Interstitial pneumonitis with alveolar exudation and interstitial infiltration were seen in all infected mice (Fig. 5), but the severity of the damages in the lungs varied remarkably between pregnant and non-pregnant mice. With wild type virus infection, mild bronchitis and foci of thickened alveolar walls with unaffected normal alveolar structure were found in non-pregnant mice (Fig. 5Aa), whereas moderately severe alveolitis with exudation, leukocytic infiltration and alveolar damage were found in pregnant mice infected by wild type virus (Fig. 5Ab). Again, it was found that much more severe necrotizing bronchitis, large areas of pulmonary consolidation with inflammatory cell infiltrates and moderate-to-severe pulmonary edema were found and were more frequently observed in infected pregnant mice (Fig. 5Ad) than in non-pregnant mice (Fig. 5Ac) infected by mutant virus.

### Pathological changes in other organs of infected pregnant mice

No significant pathological changes were observed in brains and spleens of infected mice (data not shown). Mild cell and tissue injuries, mainly in forms of cellular edema and degeneration, were found in liver (Fig. 5B) and kidney (Fig. 5C) from mice infected with the mutant virus. The most severe tissue damage was seen in mutant virus infected pregnant mice which exhibited diffuse edema, degeneration and focal necrosis of hepatocytes (Fig. 5Bd) and degeneration of renal tubular epithelial cells in kidney (Fig. 5Cd). Similar but milder liver and kidney damages were also present in wild type virus infected pregnant mice (Fig. 5Bb & Cb), but they were not observed in non-pregnant mice infected by either wild type (Fig. 5Ba & c) or mutant virus (Fig. 5Ca & c).

### Elevated levels of pro-inflammatory cytokines and chemokines in lung homogenates

**Figure 6:**
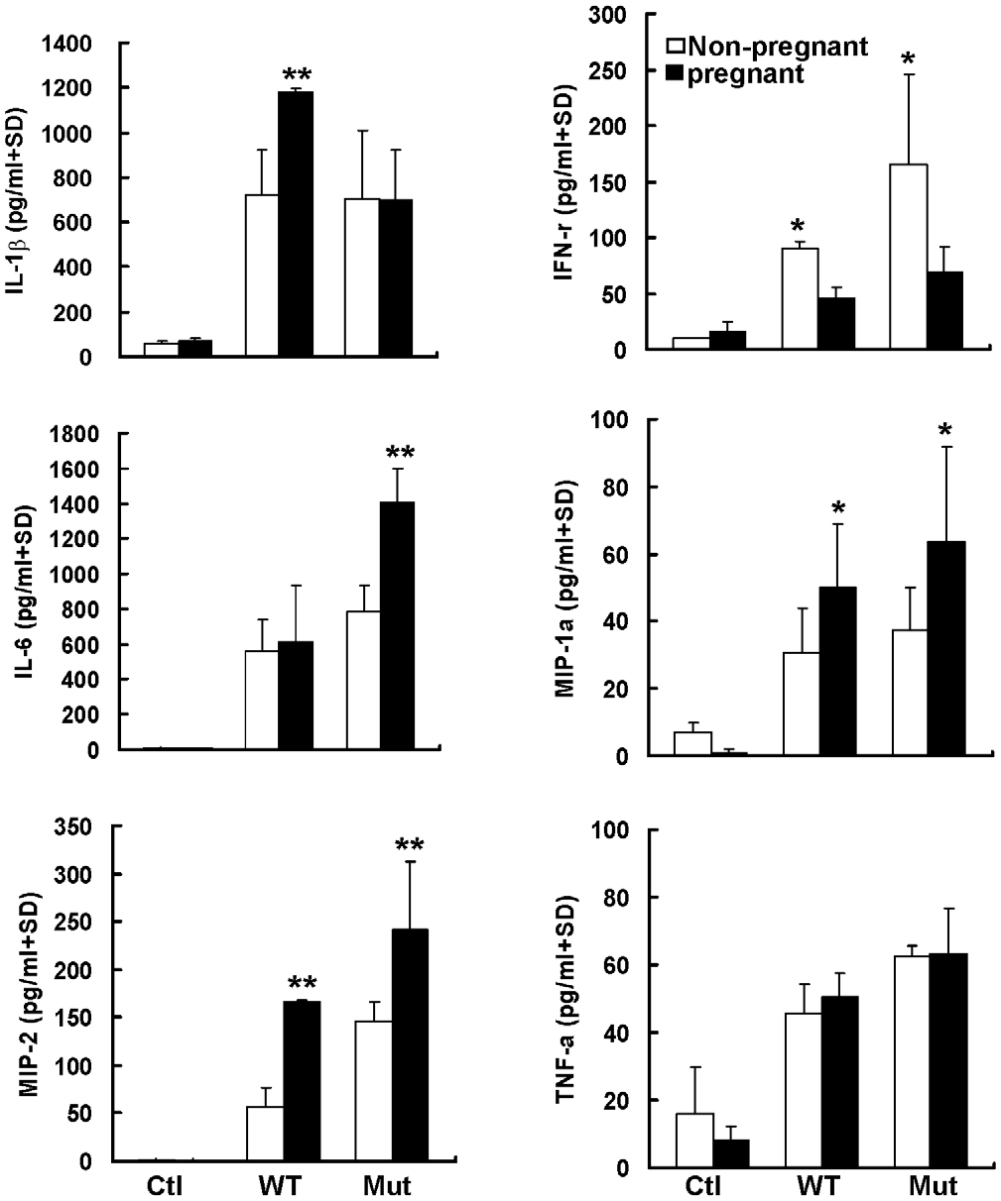
Proinflammatory cytokines and chemokines in lung homogenates. Proinflammatory cytokines and chemokines in lung homogenates collected from wild type (WT) or mutant (Mut) virus infected mice at day 3 post-infection and uninfected mice (Ctl) were detected by ELISA. ** indicates significant differences (*P*<0.001) and * indicates significant differences (*P*<0.05) between pregnant and non-pregnant mice. IL-1β: interleukin-1β, IL-6: interleukin-6, IFN-γ: interferon-γ, MIP-1α: macrophage inflammatory protein 1α, MIP-2: macrophage inflammatory protein 2; TNF-α: tumor necrosis factor-α.

To study whether the severity of pneumonitis was associated with elevated pro-inflammatory cytokines and chemokines in infected pregnant mice, levels of interleukin-1β (IL-1β), interleukin-6 (IL-6), interferon-γ (IFN-γ), macrophage inflammatory protein (MIP)-1α, MIP-2 and tumor necrosis factor-α (TNF-α) in the lung homogenates of infected mice were tested by ELISA. Elevated levels of pro-inflammatory cytokine and chemokine in the lung homogenates were observed in the virus infected mice as compared to the mock-infected control on day 3 post-infection (Fig. 6). Compared to non-pregnant mice, significantly higher levels of IL-1β, MIP-1α and MIP-2 were detected in pregnant mice infected with wild type virus (*P*<0.03), while levels of IL-6, MIP-1α and MIP-2 were elevated significantly in pregnant mice infected by mutant virus (*P*<0.02). Notably, level of IFN-γ was significantly lower in pregnant mice than in non-pregnant mice (*P*<0.05), whereas TNF-α level was similar between pregnant and non-pregnant mice, whether they were infected by wild type or mutant virus. Similar profiles of cytokine and chemokine responses to the infection were also found in serum samples collected at day 3 post-infection (data not shown).

### Impaired T cell and antibody responses in pregnant mice

**Figure 7:**
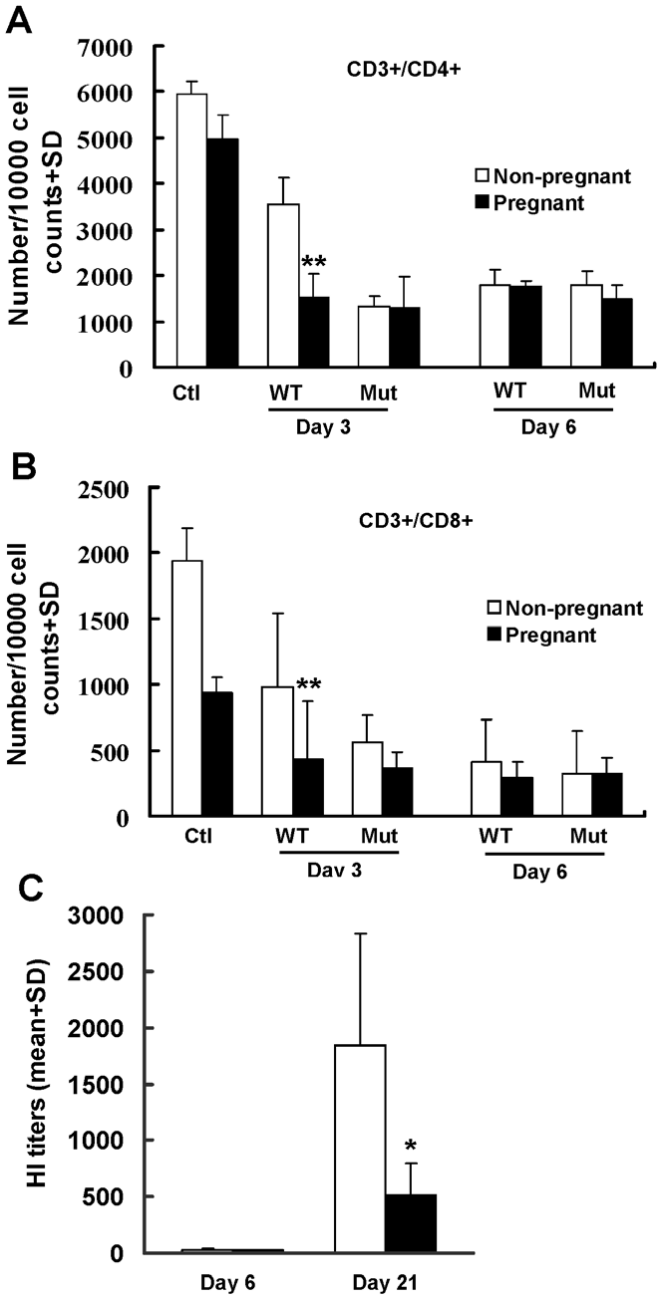
Detection of blood T cell levels and antibody response. A. CD3+/CD4+ and B. CD3+/CD8+ T lymphocytes were detected in blood samples collected from wild type (WT) or mutant (Mut) virus infected mice at indicated days post-infection and uninfected control mice (Ctl). Compared to uninfected mice, numbers of CD3+/CD4+ and CD3+/CD8+ peripheral blood T lymphocytes were significantly lower in mice either infected with wild type or mutant virus (*P*<0.0001). C. Levels of antibodies were detected in serum samples collected from infected mice at indicated days post-infection by HI assay. ** indicates significant differences (*P*<0.001) and * indicates significant differences (*P*<0.02) between pregnant and non-pregnant mice. Serum samples from 10 non-pregnant and 10 pregnant collected on day 6, and from 14 non-pregnant and 6 pregnant mice on day 21, were tested for antibody.

Compared to uninfected mice, numbers of CD3+/CD4+ (Fig. 7A) and CD3+/CD8+ (Fig. 7B) peripheral blood T lymphocytes were significantly lower in mice either infected with wild type or mutant virus (*P*<0.0001). Levels of these T lymphocytes were also significantly lower in pregnant than in non-pregnant mice infected with wild type virus at day 3 post-infection (*P*<0.001). Besides cellular immunity, specific antibody titers in survived pregnant mice were also significantly lower than that of surviving non-pregnant mice (Fig. 7C). These results suggest that both cellular and adaptive humoral immune responses are impaired in pregnant mice.

### Pathological and immunological changes in placental and fetal tissues of infected pregnant mice

**Figure 8:**
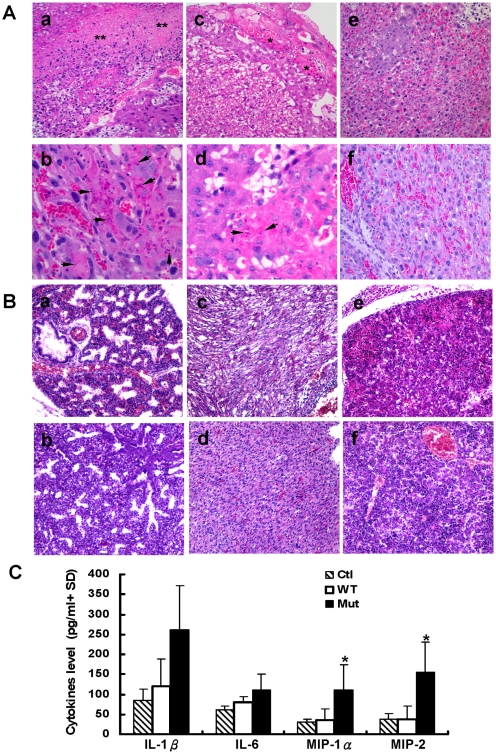
Pathological and immunological changes in placenta and fetus. A. Placentas collected from pregnant mice infected with mutant (a & b) or wild type (c & d) virus at day 3 post-infection were examined after H&E staining. a, ** indicated large area of necrosis, c, * indicated two smaller foci of necrosis in the junctional zone of the placenta. Arrows in b &d indicated apoptotic bodies and nuclear debris in the trophoblast. e & f are placentas from uninfected control mice showing no significant pathological changes. Magnification of a, c, e & f 200x and b & d 400x. B. Representative sections from fetal lung (a), myocardium (c) and liver (e) tissues from mutant virus infected mice but not obvious in wild type virus infected mice (b, d & f). Original magnification: 200×. C. Levels of indicated cytokines and chemokines in amniotic fluids collected from mice infected with wild type (WT) or mutant (Mut) virus and uninfected mice (Ctl) were detected by ELISA. * indicates significant differences (*P*<0.05) between pregnant mice infected with wild type and mutant viruses. IL-1β: interleukin-1β, IL-6: interleukin-6, IFN-γ: interferon-γ, MIP-1α: macrophage inflammatory protein 1α, MIP-2: macrophage inflammatory protein 2; TNF-α: tumor necrosis factor-α.

In mutant virus infected pregnant mice, at day 3 post-infection (corresponding to gestational date 16–18 days), patchy necrosis of placental trophoblast was found in all 13 placentas from 8 pregnant mice examined (Fig. 8Aa). Trophoblast apoptosis as evidenced by apoptotic bodies was also detected (Fig. 8Ab). Similar lesions were found at day 6 post-infection (data not shown). In wild type virus infected mice, these changes were present but mild. Smaller foci of necrosis and scattered apoptotic cells were found in 7 of the 15 placentas examined (Fig. 8Ac and d), often located in the junctional zone. However, all these histological changes were not detected in the placental tissue from non-infected control mice (Fig. 8Ae & f).

Histopathological examination of fetal tissues from pregnant mice infected with mutant virus at day 3 and day 6 post-infection showed various degrees of pulmonary congestion (Fig. 8Ba) and myocardial degeneration (Fig. 8Bc). Multiple foci of necrosis were found in fetal liver tissues (Fig. 8Be). These pathological changes were not obvious in mice infected with wild type virus (Fig. 8Bb, d & f).

Compared with uninfected pregnant mice, no significant changes of cytokine and chemokine levels was found in the amniotic fluid collected at day 3 post-infection (data not shown), whereas marked elevations of MIP-1α and MIP-2 were detected in mutant virus infected pregnant mice at day 6 post-infection (Fig. 8C). Mutant virus infection of pregnant mice also affected the development of fetus as evidenced by the smaller size of the fetus (Table 2). At day 3 post-infection (16–18 days of gestation), the average body weight of the 16 fetuses from mutant virus infected mice was 0.55 ± 0.14 g, which was significantly lighter than the average weight of fetuses (0.66 g ± 0.15 g ) from the wild type infected mice (*P*<0.05). At day 6 post-infection (gestation days 19–21), none of the mutant virus infected pregnant mice gave birth. The fetuses removed at day 6 had an average body weight of 0.77 ± 0.19 g, which was significantly lower than that of wild type infected pregnant mice (1.09 ± 0.06 g, *P*<0.001). All wild type virus infected mice gave birth at the end of gestational date and all newborns appeared normal. Furthermore, no gross morphological changes that indicated teratogenic effects on fetal development were found in the fetus from mutant virus infected mice.

**Table 2:** Effect of wild type and mutant pandemic virus infection on the development of mouse fetus.

Gestation period	16–18 days		19–21 days	
Days post-infection	Day 3		Day 6	
Virus	Mutant	Wild type	Mutant	Wild type
Fetus weight (gm)				
Mouse # 1Fetus A	0.8481	0.5135	0.9217	1.0569 (born)
Fetus B	0.7987	0.4920	1.0341	1.0825 (born)
Mouse # 2Fetus A	0.5216	0.7743	0.5756	1.1409 (born)
Fetus B	0.5648	0.4246	0.6503	1.2133 (born)
Mouse # 3Fetus A	0.6549	0.5656	0.6123	1.037 (born)
Fetus B	0.6159	0.8327	0.696	1.0705 (born)
Mouse # 4Fetus A	0.4623	0.8020	0.887 (dead)	1.0574 (born)
Fetus B	0.5553	0.5656		
Mouse # 5Fetus A	0.3447	0.6381		
Fetus B	0.3412	0.5152		
Mouse # 6Fetus A	0.4154	0.5405		
Fetus B	0.4839	0.8009		
Mouse # 7Fetus A	0.5201	0.9367		
Fetus B	0.6469	0.7987		
Mouse # 8Fetus A	0.5604	0.6613		
Fetus B	0.5179	0.6613		
**Average**	**0.5533**	**0.6577**	**0.7681**	**1.094**
**SD**	**0.1401**	**0.1499**	**0.1774**	**0.062**
***P*** ** value**	**0.0056**		**0.0003**	

## Discussion

While generally mild for most patients, the pandemic influenza A(H1N1) 2009 virus cause disproportionately more severe outcome in pregnant women. Since replication of this pandemic influenza virus can occur in placenta originated JEG-3 cells, a pregnant BALB/c challenge model was established to study the temporal virological, immunological and histopathological changes. Since differential severity caused by genetic variants of the pandemic H1N1 virus has been reported [13],[14],[15],[17],[18], we compare disease severity associated with different phenotypic strains by using one clinical isolate which causes relatively mild disease, and its mouse adapted mutant virus which is able to cause much more severe disease and even fatal outcome in mice. Challenge of pregnant mice with the same viral inoculum caused more severe interstitial pneumonitis and higher mortality than the non-pregnant controls with either wild type or mouse adapted mutant virus. These findings corroborated with higher rates of hospitalization, ICU admission for severe disease and mortality in pregnant women than the general female population [7]. As expected from findings of our previous study [19], the D222G mutant virus was also more virulent for pregnant mice. Though some previous studies suggested teratogenic effects in human [25], chick and mouse embryos exposed to influenza B [26] and reduction of cortical matter in pregnant Rhesus monkeys [27], no gross teratogenicity was observed in our cohort of mice.

A switch of T lymphocyte helper immune function from Th1 to Th2 which induces a state of relative immunosuppression to prevent rejection of the fetus during pregnancy was hypothesized [28] to explain the severe illness in human pregnancy. Indeed extrapulmonary and fetal infection was demonstrated in pregnant women infected by seasonal influenza A H3N2 and avian influenza A H5N1 viruses [29],[29,30]. Though the human placental cell line JEG-3 can be infected by this pandemic virus with increased expression of cytokine and chemokines, no evidence of extrapulmonary dissemination of virus was detected by viral culture or quantitative RT-PCR in our mouse model challenged by wild type or mouse adapted mutant with the D222G mutation. Similar to most previous studies in human pregnancy, florid inflammatory damage in the lungs was associated with marked increase in proinflammatory cytokines and chemokines in our pregnant mice model. Levels of cytokines and chemokines that mainly reflected inflammatory responses such as MIP-1α, MIP-2, IL-1β or IL-6, were significantly worse in infected pregnant mice, while those markers indicating not only inflammatory but also antiviral immune responses such as TNF-α and IFN-γ, were similar or even significantly lower in pregnant mice as compared to non-pregnant mice. The spillover of some of these pro-inflammatory cytokines or chemokines from the lungs was evident paralleled by similar increase in their levels in the serum and amniotic fluid. Thus the involvement of the placenta and fetus as evident by placenta cell necrosis and apoptosis, and fetal tissue degeneration were just manifestations of the severe systemic inflammatory response syndrome and hypoxia due to respiratory failure rather than the effect of extrapulmonary viral replication and cytolysis. Indeed no isolation of pandemic virus was reported from human placental or fetal specimens up to this time. Another important determinant of severity in human influenza infection is the inability of the host to control the viral load [3],[31]. Previous study showed a lower serum IgG_2_ in pregnant women affected by severe pandemic influenza [32]. In our study, the highest viral load and mortality were found in pregnant mice infected with mutant virus which was associated with lower peripheral blood T lymphocytes and hemagglutination inhibitory antibody titer.

The higher virulence of mouse adapted mutant virus in both pregnant and non-pregnant mice than the wild type virus were explained by the turkey erythrocyte binding assay which showed that the mouse adapted virus with D222G mutation has a much stronger avidity of binding to sialic acid with 2,3 linkage to galactose. Such 2,3-linked sialic acid receptors are highly abundant in the upper and lower respiratory tree of mice [33] and relatively abundant in the lower respiratory tree of human. The finding corroborated with the higher rate of detection of this mutant in severe cases of pandemic influenza 2009 in human and especially in their lower respiratory tract specimens. Though seasonal influenza A virus tends to infect type I pneumocyte in human, this pandemic virus tends to infect type II pneumocyte in our mouse model. The significance of this finding remains to be determined.

In summary, the detrimental effect of pandemic influenza virus in pregnant mice mainly relies on the viral replication and associated inflammation in the lungs which increased proinflammatory cytokine and chemokines in the systemic circulation and amniotic fluid with no evidence of extrapulmonary viral dissemination in our model even when a D222G mouse adapted mutant virus was used.
